# The neurochemistry and social flow of singing: bonding and oxytocin

**DOI:** 10.3389/fnhum.2015.00518

**Published:** 2015-09-23

**Authors:** Jason R. Keeler, Edward A. Roth, Brittany L. Neuser, John M. Spitsbergen, Daniel J. M. Waters, John-Mary Vianney

**Affiliations:** ^1^Brain Research and Interdisciplinary Neurosciences Laboratory, School of Music, Western Michigan UniversityKalamazoo, MI, USA; ^2^Department of Biological Sciences, Western Michigan UniversityKalamazoo, MI, USA

**Keywords:** singing, oxytocin, social flow, ACTH, improvisation, music, bonding, trust

## Abstract

Music is used in healthcare to promote physical and psychological well-being. As clinical applications of music continue to expand, there is a growing need to understand the biological mechanisms by which music influences health. Here we explore the neurochemistry and social flow of group singing. Four participants from a vocal jazz ensemble were conveniently sampled to sing together in two separate performances: pre-composed and improvised. Concentrations of plasma oxytocin and adrenocorticotropic hormone (ACTH) were measured before and after each singing condition to assess levels of social affiliation, engagement and arousal. A validated assessment of flow state was administered after each singing condition to assess participants' absorption in the task. The feasibility of the research methods were assessed and initial neurochemical data was generated on group singing. Mean scores of the flow state scale indicated that participants experienced flow in both the pre-composed (*M* = 37.06) and improvised singing conditions (*M* = 34.25), with no significant difference between conditions. ACTH concentrations decreased in both conditions, significantly so in the pre-composed singing condition, which may have contributed to the social flow experience. Mean plasma oxytocin levels increased only in response to improvised singing, with no significant difference between improvised and pre-composed singing conditions observed. The results indicate that group singing reduces stress and arousal, as measured by ACTH, and induces social flow in participants. The effects of pre-composed and improvised group singing on oxytocin are less clear. Higher levels of plasma oxytocin in the improvised condition may perhaps be attributed to the social effects of improvising musically with others. Further research with a larger sample size is warranted.

## Introduction

People often report a feeling of connectedness during music experiences, either as a listener or a performer. Musicians often discuss “feeling lost in the music” or “finding the groove” during improvisatory experiences (e.g., “trading fours” in a jazz performance) and audience members frequently share this sense of cohesion through a commitment to the music (Pitts, 2004; Hytönen-Ng, 2013). The colloquial phrases used to describe this engagement and connectedness within music experiences align with the theoretical construct of flow, which is an optimal psychological state in which a person is completely absorbed in the task at hand (Csikszentmihalyi, 1975). When experienced in group settings, flow holds the potential to facilitate social connection, but the present research base examining social flow and interpersonal connection in music experiences is limited (Hart and Di Blasi, 2015). Additionally, little is known about the neurochemical processes that facilitate social bonding during group music experiences. Over the past two decades, neuroscience research in music has relied heavily on neuroimaging, mapping regions of the brain active during music production and perception. It is only more recently that the neurochemical responses to music have been investigated. Chanda and Levitin (2013) review the chemical and biological effects of music and express a strong need for further research. Current evidence suggests that music's effects on health and well-being may be modulated through engagement of neurochemical systems (Chanda and Levitin, 2013; Fancourt et al., 2014). In particular, group singing has demonstrated positive effects on emotional states and biological outcomes, implicating the neuroendocrine system as a potential underlying mechanism (Kreutz et al., 2004; Kreutz, 2014; Fancourt et al., 2015). The neuropeptide oxytocin may in part be responsible for the social and health benefits of music, while adrenocorticotropic hormone (ACTH) may mediate the engagement and arousal effects of music (Chanda and Levitin, 2013; Kreutz, 2014). These physiological processes may consequently influence the subjective experience of social flow and perception of social connection during music experiences.

### Oxytocin and ACTH

Oxytocin is a neuropeptide produced by large neuroendocrine cells of the supraoptic and paraventricular nuclei of the hypothalamus. Oxytocin is transported from the large neuroendocrine cells to the posterior lobe of the pituitary gland, where it is subsequently released into the bloodstream as a hormone. The paraventricular nucleus (PVN), where oxytocin synthesis is most concentrated, coordinates signals from the brain in response to stress and controls the hypothalamic-pituitary-adrenal (HPA) axis (Herman, 2012). Neurons in the PVN release corticotropin releasing-factor (CRF), which promotes the secretion of ACTH into peripheral circulation. ACTH is a neurohormone that stimulates the synthesis and release of glucocorticoids, such as cortisol, from the adrenal gland (Grossman et al., 1982). Oxytocin is colocalized with stress hormones in the PVN and has suppressive effects on the HPA axis, including ACTH (Gibbs, 1986; Windle et al., 2004; Carter, 2014).

The word oxytocin is derived from the Greek words meaning “quick birth.” In humans, functions of oxytocin were originally associated with maternal behaviors such as mother-infant bonding, breast-feeding, and uterine contractions (Takahashi et al., 2013). More recent findings reveal the broader scope of oxytocin in human social and emotional behaviors, with effects that are highly dependent on context and individual traits (Bartz et al., 2011). Oxytocin mediates social behavior (Heinrichs et al., 2009) and regulates stress and anxiety (Ditzen et al., 2009). Depending on the context and the individual, it is hypothesized that oxytocin may elicit positive or negative social emotions (Bartz et al., 2011). Under optimal circumstances, oxytocin increases trust (Kosfeld et al., 2005) and is associated with a parent's social attachment to their children (Feldman et al., 2010). While oxytocin production in humans was originally believed to increase only in response to direct physical contact, mothers bonding with their infants demonstrated higher plasma oxytocin levels from vocalizations alone (Leslie et al., 2010). The extended period of nurturing facilitated by oxytocin, as well as it's role in reproductive behavior and physiologic functions, indicate it's importance in human social and intellectual development (Carter, 2014). Research linking social behaviors to positive health and disease outcomes implicates oxytocin as a primary physiologic mechanism (Uchino, 2006).

ACTH may mediate the engagement and arousal effects of music (Chanda and Levitin, 2013). ACTH, which mediates attention (Sandman et al., 1975, 1977) and distress (Mauri and Volpe, 1994), responds to various types of challenges or pain perceived in higher levels of the brain (Herman, 2012). While there appears to be a general consensus among studies that music listening enhances oxytocin synthesis, the role of ACTH is less clear. Preliminary evidence suggests that listening to stimulating music, such as techno, increases plasma ACTH while relaxing music reduces ACTH synthesis and circulation (Gerra et al., 1998). ACTH is examined in this study because it responds to stimuli in seconds (Weijnen and Slangen, 1970). The short duration of each singing condition in this study indicate that ACTH may be implicated in behaviors of arousal and attention. To date, no studies were found that examined both ACTH and oxytocin in active music production.

### Social affiliation and engagement in music

A handful of studies have examined endogenous oxytocin during music production and perception. Postoperative patients listening to relaxing music through headphones demonstrated an increase in serum oxytocin and reported higher levels of relaxation compared to a control group with no music (Nilsson, 2009). Choral singing has been shown to increase salivary oxytocin and elicit positive emotional states (Kreutz et al., 2004; Kreutz, 2014). In professional and amateur singers, peripheral oxytocin increased after an individual 45 min singing lesson, however, music parameters were not identified and non-musical interactions during the lesson may have influenced outcome measures (Grape et al., 2002). In that same study, amateur singers demonstrated a decrease in post-singing levels of cortisol, while professional singers showed the opposite trend, pointing toward higher levels of perceived stress and arousal in the professional singers. This trend is consistent with the recent findings of Fancourt et al. (2015), where low-stress singing without an audience reduced levels of salivary cortisol and cortisone, and high-stress singing in front of a large audience increased levels of both glucocorticoids. Depending on individual traits and context, it is possible that singing may be perceived as a stressful experience with corresponding biological responses. In general, singing appears to have potential benefits on psychological health and well-being, with additional indications of potential physical benefits (Clift et al., 2010). More research is needed, however, to identify the underlying mechanisms linking singing to physical and psychological health.

While there is little information on the neurochemistry of singing, previous research has demonstrated the effects of singing on behavioral and self-reported outcomes. Group singing produced the highest scores on trust and cooperation compared to other group activities, as measured by a trust and dilemma game (Anshel and Kipper, 1988). In those with mental illness, singing has been found to increase mental health, well-being, and social skills (Clift and Morrison, 2011). Children's sense of inclusion and belonging with their peers was positively correlated with their singing abilities in a longitudinal study on the social impact of music (Welch et al., 2014). This falls in line with the theory that music has evolved as a means of social bonding, with evolutionary roots in parent-infant attachment (Freeman, 1998). Therefore, the belief that oxytocin plays a large role in the social and health benefits of music appears to be supported by previous behavioral findings.

### Social flow

The concept of flow, frequently referred to as flow state, was first introduced to the field of positive psychology in 1975 by Csikszentmihalyi. According to Csikszentmihalyi, to experience flow is to experience an optimal psychological state (1975). People experiencing flow find themselves completely immersed in the present activity, so intensely focused that all unrelated thoughts and emotions seemingly disappear from their conscious being, allowing for efficient yet effortless execution of thoughts and actions. Autotelic in nature, the flow experience is both enjoyable and intrinsically rewarding (Csikszentmihalyi, 1975, 1997). Though the primary focus of flow theory lies in the flow experience itself, positive consequences of flow including increased motivation, creativity, efficacy, and subjective well-being have been observed (Csikszentmihalyi and LeFevre, 1989; Jackson et al., 2001; Mugford, 2004; Fritz and Avsec, 2007; Engeser, 2012; Salanova et al., 2014).

In recent years, researchers have expressed a growing interest in the concept of social flow, which involves not only optimal performance, but also optimal interaction with others (Csikszentmihalyi, 1975; Bachen and Raphael, 2011; Engeser, 2012). Social flow has been studied in various contexts including athletic, occupational, and familial environments. Though none of these areas have been extensively researched, the literature currently suggests that frequency of social flow experience is positively correlated with the quality of interpersonal relationships (Rathunde, 1997; Bakker et al., 2011; Salanova et al., 2014). Underlying the neurochemistry of social flow may be oxytocin and ACTH, which we explore in this study.

### Social flow and engagement in music

The literature also indicates that various music-related tasks such as music listening, composition, and performing are conducive to facilitating solitary and social flow. Colloquial phrases such as “feeling the groove” and “lost in the music” frequently arise in everyday conversation amongst musicians. Current evidence suggests that these phrases may actually be related to flow states experienced within a musical context (MacDonald et al., 2006; Baker and MacDonald, 2013; Diaz, 2013; Wrigley and Emmerson, 2013). One of the most frequently mentioned musical genres in music-related flow literature is jazz. Jazz musicians frequently report feelings associated with flow experiences such as intense oneness with their musical product and the merging of individual musicians to form a single cohesive entity when performing, particularly when improvising and exploring new sounds (Hytönen-Ng, 2013).

Walker (2010) explored differences between flow experienced in low-interdependent and high-interdependent tasks in the context of a singles and doubles racket sport game. He found that, though the number of consecutive volleys did not differ across conditions, the high-interdependent task was rated as more challenging and the flow experience was reported to be more intense than in the low-interdependent task. In a musical context, participation in a standard performance of a pre-composed piece may be considered a low-interdependent task whereas playing or singing in an improvisatory manner can be viewed as a high-interdependent task. Improvisation requires clear communication and cooperation from all group members. Each individual member is challenged to use both their technical competence and artistic instinct in creating an innovative sound within a given musical structure, maintained by the group as a whole (Sawyer, 2006; Rogers, 2013). To date, no experimental studies have been found that examined flow in the context of group music improvisation.

As a feasibility study, a primary purpose was to evaluate the design and methodology. In a recent review, LaGasse (2013) highlights the importance of pilot and feasibility studies in music therapy, especially in previously unstudied areas. The appropriateness of the methodology was assessed by utilizing a small sample size and previously validated data collection procedures. It is hoped that preliminary findings from this study will inform future researchers on the feasibility of neurochemical and flow state data collection in vocal music production. We compared a standard, pre-composed singing performance with an improvised performance of the same song, and hypothesized that vocal improvisation would elicit higher measures of social flow and bonding when compared to a pre-composed vocal performance.

## Methods

### Participants

Four participants (2 males and 2 females) were conveniently sampled based on the following inclusion criteria: jazz vocalists, students at a large Midwestern American university, and over 18 years of age. We included jazz vocalists to explore the neurochemistry and social flow of vocal improvisation within a group context. Vocal quartets are common in jazz music, and therefore, the group structure was familiar to participants and limited extraneous challenges in a controlled setting. Participants were excluded from the study if they met any of the following criteria: medical or psychiatric illness, smoking more than 15 cigarettes per day, drug or alcohol abuse, weighing less than 110 lbs., bleeding disorders (e.g., hemophilia), and pregnancy. Participants were asked to abstain from food and drink (other than water) 2 h before the experiment and from smoking, caffeine, and alcohol 24 h before the experiment.

### Design

**Figure 1:**
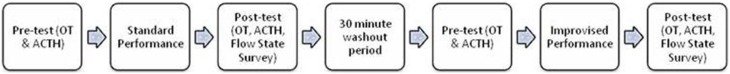
**Study design**.

A mixed design using repeated measures was utilized to explore the effects of pre-composed and improvised group singing on social flow and neurochemical measures of connectedness and arousal (see Figure 1). Participants formed one group and performed the same song together in two conditions. The first condition was a performance of the music as it was written without improvisation or further embellishment of the melody, referred to from this point forward as “standard performance” (SP). The second condition was a performance of the music that followed the syntactical harmonic structure (chord changes) of the composition, with improvised melodies, referred to from this point forward as “improvised performance” (IP). In each condition, pre and post-tests measured plasma oxytocin and ACTH, and a post-test survey assessed the level of social flow experienced by participants. Based on the brief duration of each performance and the short half-life of plasma ACTH and oxytocin, a 30 min washout period was utilized between conditions to allow neuropeptide levels to return to baseline. The reported half-life of plasma ACTH is approximately 10 min (Yalow et al., 1964). Similarly, the half-life of plasma oxytocin is estimated at 5–10 min (Amico et al., 1987). In healthy subjects, oxytocin levels peaked after only 5–8 min of music listening, with plasma levels returning toward baseline after 7–10 min (Dai et al., 2012). In a different study examining music perception, researchers utilized a 10 min washout period prior to obtaining baseline measures of plasma ACTH (Evers and Suhr, 2000). Therefore, it was estimated that a period of 30 min between conditions would allow neuropeptide levels to return to baseline. This study was reviewed and approved by the Human Subjects Institutional Review Board at Western Michigan University. Informed written consent was obtained from all participants involved in the study.

### Musical considerations

All musical decisions were made in collaboration with the university's school of music vocal jazz director, who was familiar with the participants' skill level and repertoire. The jazz standard “Centerpiece” (Edison and Hendricks, 1958) served as the musical content for both the standard and improvised conditions. The vocal jazz director created two vocal quartet arrangements of the piece, one for the standard performance condition (SP) and one for the improvised performance condition (IP). The SP arrangement was sung as written, with no improvisation or embellishments. The IP arrangement began with the unison singing of the original melody and then allowed time for each participant within the group to improvise over the basic harmonic structure of the original song.

### Blood draws

Six milliliters of blood was drawn from the antecubital vein into 6 ml EDTA lavender top tubes containing 5.0 mg EDTA and 2.500 KIU aprotonin. A sterile field was maintained using a Vacutainer blood draw kit. Whole blood was immediately placed on ice and transported to the adjacent lab, where it was centrifuged at 1200 rpm for 12 min at 4°C. Six milliliters of whole blood yielded approximately 2 ml of plasma per participant. Plasma was aliquoted into microtubes and stored at −70°C until analysis.

### Measures

Oxytocin and ACTH concentrations were determined using enzyme-linked immunosorbent assays kits produced by Enzo Life Sciences, Inc. (Farmingdale, NY, USA). The oxytocin kit has been previously validated for human plasma using various methods, including mass spectrometry (Carter et al., 2007). Sensitivity for oxytocin and ACTH was 15.0 pg/mL and 0.46 pg/mL, respectively. All samples were run in duplicate. Oxytocin plasma samples were diluted 1:8 with assay buffer and run unextracted. Both assays were run according to manufacturer instructions. Due to the small sample size, only one plate was needed to analyze each neuropeptide. The intra-assay coefficients of variations for ACTH and oxytocin were 15 and 9%, respectively. All tests were performed in collaboration with the Department of Biological Sciences at Western Michigan University.

There is controversy surrounding the measurement of oxytocin on unextracted samples. In human fluids, there may be interference from various proteins and other substances leading to unreliable measurements (McCullough et al., 2013). However, it is also argued that extraction protocols may underestimate peripheral oxytocin concentrations, as a majority of oxytocin is lost during the extraction process (Martin and Carter, 2013). To avoid precipitation of oxytocin in the blood, samples were run unextracted using a previously validated, sensitive, and specific commercially-available kit.

Social flow was measured using the Flow State Scale-2 (FSS-2; Jackson et al., 2010), a 36-item questionnaire that assessed individual's perceived level of flow within a specific event. The FSS-2 is a post-event assessment and was administered immediately following post-test blood draws in each condition. The 36 items (questions) in the FSS-2 reflect Csikszentmihalyi's definition of flow, with four items directly addressing each of the following nine flow dimensions: Challenge-skill balance, merging of action and awareness, clear goals, unambiguous feedback, concentration on the task at hand, sense of control, loss of self-consciousness, transformation of time, and autotelic experience. Participants were directed to respond to each statement using a 5-point Likert scale in which a score of 1 indicated “Strongly Disagree” and a score of 5 indicated “Strongly Agree.” The FSS-2 was scored by first calculating the total raw score for each dimension. Each raw dimension score was then divided by 4 to compute the average dimension score. The sum of all average dimension scores provides the total scale score. Group means for each dimension and total scaled scores were used to determine the level of social flow experienced. The maximum total scale score of 45 signifies the ultimate flow experience. The minimum total scale score of 9 signifies no flow. The FSS-2 has demonstrated strong construct validity and reliability across various physical activities including yoga, basketball, soccer, running, and football (Jackson and Eklund, 2002; Jackson et al., 2008). The FSS-2 has also been shown to be valid and reliable in measuring flow during live music performance, with Cronbach's alpha ranging from 0.81 to 0.92 for each of the nine dimensions (Wrigley and Emmerson, 2013).

### Procedure

Participants (*n* = 4) formed one group together (a vocal quartet). At the beginning of the experiment, the consent form was reviewed and participants received a brief overview of the procedures. Participants were instructed to avoid physical contact during the experiment. SP pre-test blood draws were then conducted for two participants at a time in a separate room. The remaining two participants received the SP pre-test blood draw after each previous participant was finished. Given the labile nature of oxytocin and ACTH, two phlebotomists were used to expedite the blood collection process and minimize potential protein breakdown after whole blood was placed on ice. Following the SP pre-test blood draws, the vocal jazz director provided participants with brief musical instructions lasting approximately 5 min. Participants were instructed to sing their respective part of the music as it was written, without any embellishment or improvisation. Immediately following the instructional period, participants performed the standard piece together as it was written, with accompaniment provided on the piano by the aforementioned vocal jazz director. Immediately following the standard performance, which lasted 5 min and 38 s, participants were called in pairs to the separate room where individual post-test blood draws were conducted. All participants confirmed that they were able to proceed without ill effects from the blood draws and were then escorted individually to nearby but isolated rooms where they were provided with a paper copy of the FSS-2 and a pencil. Each individual was instructed to complete the FSS-2 according to the directions located at the top of the test-page and upon completion of the survey, take time to rest before completing the second performance. Following the 30 min test-and-rest period, surveys were submitted and individual pre-test blood draws for the improvised performance were conducted. Following the same format as the SP condition, participants received 5 min of instructions prior to the improvised performance. Participants performed the improvised piece together with extensive embellishment and improvisation. The duration of the improvised performance was 6 min and 1 s. Immediately after the improvised performance, individual blood draws were conducted and participants were again given the flow-state survey to complete in separate rooms. Following completion of the surveys, participants were provided with follow up information and thanked for their time.

### Results

Here we present initial data on social flow, oxytocin and ACTH in standard and improvised group singing. The successful implementation of procedures and collection of data indicate the feasibility of methods used in this study, which serve as our primary outcomes. Given the small sample size, this study was not sufficiently powered for statistical analyses. As such, the statistical analyses presented here are primarily for demonstrative purposes, to inform future research in this area on possible data trends and analyses. Descriptive and parametric statistical analyses were performed using SPSS (IBM) version 22.

### FSS-2

Raw FSS-2 scores indicate that participants experienced social flow in both the standard and improvised conditions. The mean total scaled score in the SP condition, with a maximum possible score of 45, was 37.06 (*n* = 4). The mean total scaled score in the IP condition, with a maximum possible score of 45, was 34.25 (*n* = 4). Mean scores for each dimension of flow for the SP and IP conditions are displayed in Table 1.

**Table 1:** **FSS-2 Dimension scores in standard and improvised conditions**.

**FSS-2 dimension subscale**	***n***	**Minimum**	**Maximum**	**Mean**	**Std. deviation**
**STANDARD PERFORMANCE**					
Challenge-skills balance	4	3.25	4.00	3.69	0.31
Merging action-awareness	4	3.75	4.50	4.18	0.31
Clear goals	4	3.75	4.75	4.18	0.43
Unambiguous feedback	4	3.75	4.75	3.56	0.66
Concentration on the task	4	3.25	4.25	3.94	0.47
Sense of control	4	4.00	5.00	4.63	0.43
Loss of self consciousness	4	3.25	4.50	3.88	0.52
Transformation of time	4	3.25	4.50	3.69	0.59
Autotelic experience	4	3.25	5.00	4.44	0.83
**IMPROVISED PERFORMANCE**					
Challenge-skills balance	4	3.50	5.00	4.44	0.66
Merging action-awareness	4	2.50	5.00	3.44	1.13
Clear goals	4	2.50	4.25	3.44	0.72
Unambiguous feedback	4	3.00	4.25	3.56	0.66
Concentration on the task	4	4.25	5.00	4.56	0.31
Sense of control	4	3.75	4.00	3.94	0.12
Loss of self consciousness	4	2.00	3.50	2.56	0.66
Transformation of time	4	3.25	4.50	3.69	0.55
Autotelic experience	4	4.25	5.00	4.69	0.38

### Oxytocin and ACTH

**Figure 2:**
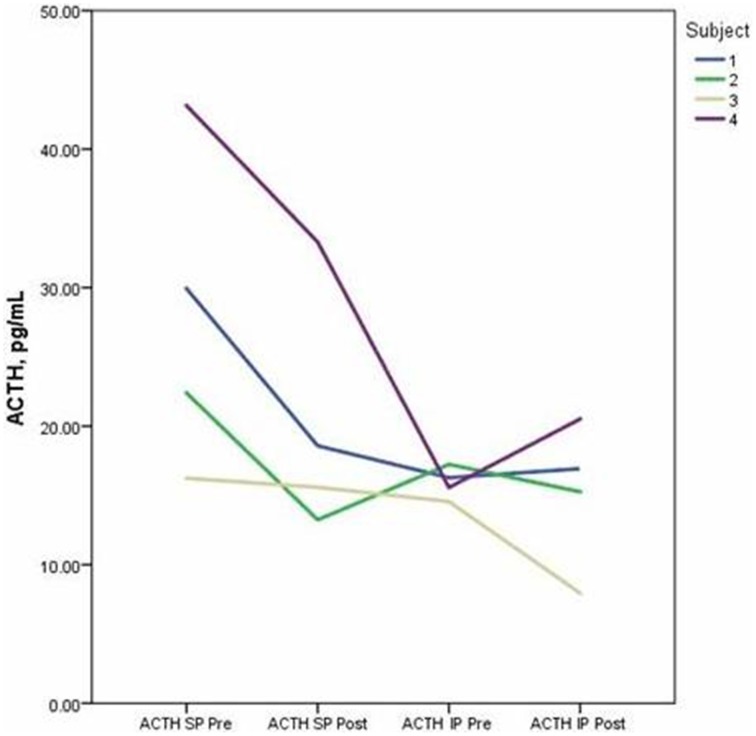
**The figure depicts individual concentrations of plasma adrenocorticotropic hormone (ACTH) at each time point measured during the standard performance (SP) and improvised performance (IP)**.

**Figure 3:**
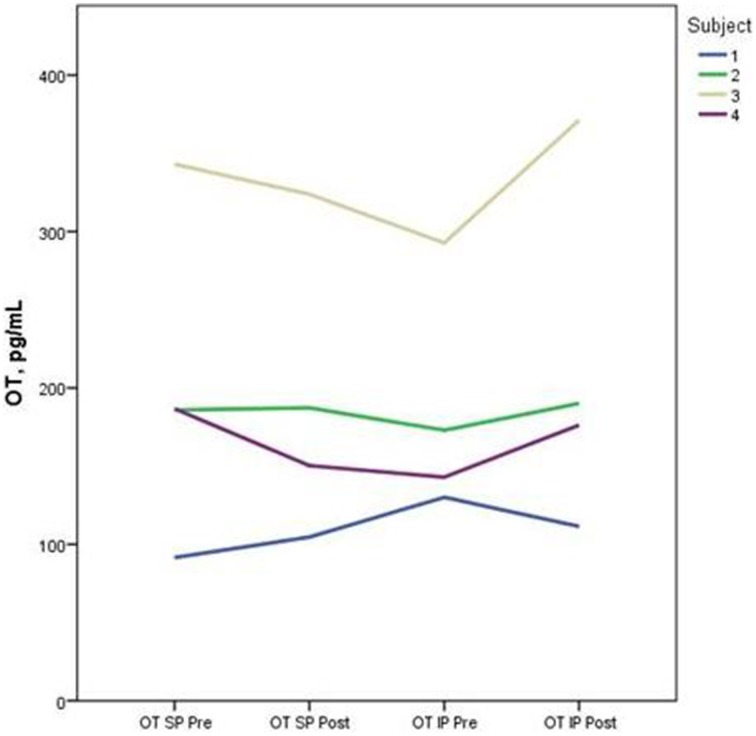
**The figure depicts individual concentrations of plasma oxytocin (OT) at each time point measured during the standard performance (SP) and improvised performance (IP)**.

**Figure 4:**
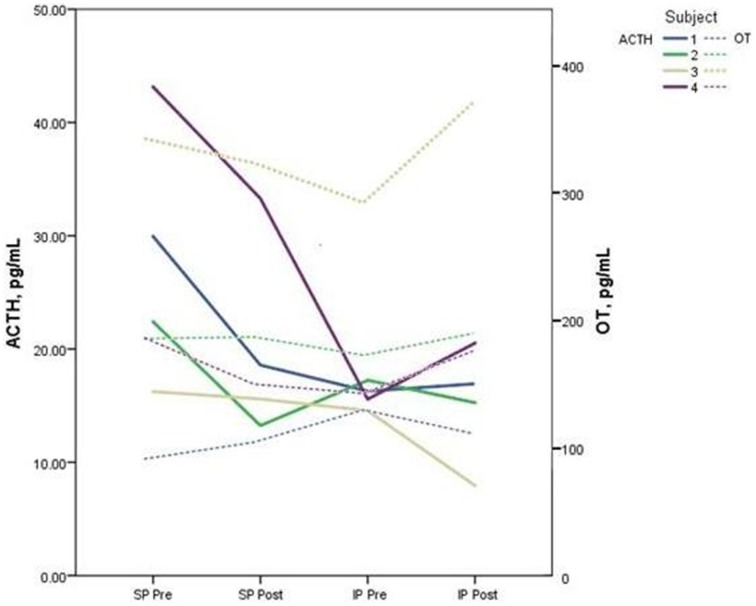
**The figure depicts the relationship between plasma adrenocorticotropic hormone (ACTH) and oxytocin (OT) concentrations for each subject measured during pre and post-tests of the standard performance (SP) and improvised performance (IP)**. An inverse relationship between neuropeptide levels is demonstrated in 3 out of 4 subjects.

Descriptive data for oxytocin and ACTH collected at each time point is presented in Table 2. Both the standard and improvised conditions revealed a mean decrease in plasma ACTH after participants sang together. The change in ACTH in the SP condition pre-to-post test (*p* < 0.05) was 21% greater than the pre-to-post test ACTH outcomes for the IP condition (*p* > 0.05). Figure 2 depicts individual changes in ACTH at each time point. Student's *t*-test of oxytocin concentrations in the SP condition revealed a mean decrease of 10 pg/mL, while the IP condition demonstrated a mean increase of 27 pg/mL. Figure 3 depicts individual changes in oxytocin concentrations across all time points. In Figure 4, individual changes in oxytocin and ACTH are compared at each time point to reveal an inversely proportional trend in 3 out of 4 subjects.

**Table 2:** **Descriptive statistics of neurochemical variables, adrenocorticotropic hormone (ACTH), and oxytocin, at each time point in the standard (SP) and improvised (IP) conditions (*n* = 4)**.

**Variable (pg/mL)**	**SP pre-test**			**SP post-test**			**IP pre-test**			**IP post-test**		
	**M**	**Med**	**SD**	**M**	**Med**	**SD**	**M**	**Med**	**SD**	**M**	**Med**	**SD**
ACTH	27.3	26.1	11.6	20.2	1 7.1	9.0	15.9	15.9	1.1	15.2	16.1	5.3
Oxytocin	201.8	186.3	104.2	191.5	168.8	94.5	184.6	157.9	74.2	212.2	183.1	111.4

## Discussion

### Feasibility of experimental procedures

The primary purpose of this study was to evaluate the feasibility of experimental research procedures that were designed to investigate the neurochemical processes and social flow experiences associated with group singing. The successful implementation of procedures and data collection indicates the feasibility of the methods employed in this study, which may help future researchers in exploring the relationships between music improvisation, neurochemical activity, and social flow experiences.

Though the drawing of blood in between the completion of the task and the distribution of the FSS-2 is a potential confounding variable in regards to the assessment of flow state, the inclusion of this element allowed the researchers to evaluate the feasibility of efficiently and effectively gathering both self-report and neurochemical data within a single condition. Based on FSS-2 scores and participant responses during the debriefing period, the procedures were effective in facilitating flow for student musicians and the blood draw did not appear to affect the subjective reports of flow. It is also important to acknowledge that seven out of the eight flow scales were completed within 15 min of the end of the task in question, as it is recommended that the FSS-2 be administered as soon as possible following participation in the activity being assessed (Jackson et al., 2010).

One participant required additional time during the final blood draw, as the phlebotomist was unable to access the antecubital vein upon the first few attempts. While the blood draw was successful after several minutes, there are inherent challenges in drawing blood from human subjects, such as syncope, that are best controlled for with a larger sample size. We were able to determine, however, that group singing was not impeded by the controlled environment and data collection procedures.

### Social flow in music improvisation

Raw FSS-2 scores indicate that social flow was experienced in both the standard and improvised conditions. Raw scores also indicate that the experience of flow was slightly greater during the standard performance than in the improvised performance. Previous research has found that creative demand within a task is positively correlated with level of flow (Baker and MacDonald, 2013). In the present study, the improvisation task was structured in a way that required more creative input than the standard performance, but did not appear to facilitate deeper flow experiences.

One particular dimension of flow, loss of self-consciousness, appeared to contribute most to the difference in flow experience between conditions as the mean dimension score was 1.32 points lower on the 5-point Likert scale during improvisation than during standard performance. This observation aligns with the findings of Wrigley and Emmerson (2013), who found that the loss of self-consciousness dimension received the lowest mean score in comparison to the other eight flow dimensions in undergraduate vocal students. While the creative demands in improvisation tasks may appear more conducive to social flow from a challenge-skills perspective, it must also be recognized that creative output in group settings requires a certain level of self-disclosure amongst group members, which may elicit anxiety and inhibit flow. The decrease in ACTH concentrations after singing in both conditions indicates a potential relationship between flow state and stress, which warrants further investigation.

In addition to the increased levels of self-disclosure in improvisation, the participants' level of flow may have been affected by their skill level and past musical experiences. For example, one participant, during debriefing discussion, mentioned that she did not improvise as frequently as the other participants and she felt unsure of herself. However, when presented with a follow up question, she stated that she did not feel as anxious in this setting as in past experiences. This may be, in part, because participants were repeatedly told that the researchers' primary focus was on the nonmusical components rather than the quality of the musical product.

Another participant mentioned that he found himself having to put forth more effort during the improvisation, stating, “When it was spontaneous, I actually had to do a lot more thinking.” This statement conflicts with previous reports of professional jazz musicians, who state that when they are improvising they frequently get “in the musical zone” and actions and sounds seem to naturally occur with little felt effort. Some professionals also describe their most memorable improvisation experiences as being surreal (Hytönen-Ng, 2013). This inconsistency between studies sheds light on the importance of accounting for the skill level and past experiences of musicians, as the experiences of student musicians such as those who participated in this study, may differ from those of professional and more experienced musicians.

### Oxytocin and ACTH

We hypothesized that group singing in both conditions would decrease stress and arousal, as measured by ACTH, and increase social bonding, as measured by oxytocin. Despite the small sample size, there was a significant decrease in pre-to-post levels of ACTH during the standard singing performance. This is consistent with previous literature demonstrating the positive effects of music on stress and the immune system (Bittman et al., 2001; Chanda and Levitin, 2013). Singing has been shown to reduce cortisol levels depending on context and individual traits, and is generally associated with psychological health and well-being (Grape et al., 2002; Clift et al., 2010; Fancourt et al., 2015). This is the first time that ACTH has been examined during active music production, as opposed to music listening, and the results appear to be supported by previous findings. The improvised singing performance demonstrated a minor decrease in ACTH from pre-to-post levels, which perhaps may be attributed to low concentrations observed at the IP pre-test. This may have been caused by carry-over effects from the first condition (see recommendations for future research).

As expected, mean concentrations of oxytocin increased during the improvised condition. Vocal improvisation naturally elicited behaviors conducive to social bonding, such as listening, responding, spontaneous communication, eye contact, and cooperation. Surprisingly, the standard performance demonstrated a mean decrease in oxytocin concentrations. This should be interpreted with caution due to the small sample size and influence of individual traits on plasma oxytocin levels (Bartz et al., 2011). Studies with a larger sample size have demonstrated significant increases in peripheral oxytocin after choral singing and individual singing lessons (Grape et al., 2002; Kreutz, 2014). The high variability of oxytocin concentrations observed among participants in the present study can be addressed through a larger sample size. A sufficiently powered study using the same design as the current study (1 – β = 0.80, effect size > 0.25, alpha < 0.05) would require 82 participants based on our data.

### Limitations

Several limitations need to be considered when interpreting the results of this study. The small sample size provides initial data, however, it does not provide enough power to make strong statistical inferences. Prior to conducting the study, participants were informed that the researchers were investigating differences between standard and improvised vocal performance. This knowledge may have influenced responses either during or following each task.

### Conclusion and recommendations for future research

This study provided support for the feasibility of conducting experimental flow research in which the structure of a musical task is manipulated. Because the present study involved a very small sample, it is recommended that a study be conducted with a larger sample size to further evaluate the experimental procedures and to gain sufficient power that would allow for effective statistical analysis of results. To avoid potential carry over effects, a longer washout period between conditions is recommended. Although previous studies indicated only a short washout period was necessary, mean plasma ACTH at baseline was significantly lower in the second (IP) condition. This may have also been attributed to an order effect, as all participants sang in the IP condition following the SP condition. A repeated measures design with counterbalancing may control for any order effect observed in this study. A washout period at the beginning of the experiment may also reduce variations in neuropeptide levels caused by pre-experimental stimuli. Given the recent findings of Fancourt et al. (2015), a potential extension of this study may also include a rehearsal period and self-report data to evaluate the level of stress experienced by participants during standard and improvised singing. In the present study, the significant decrease in ACTH during the standard performance may indicate a low-stress condition experienced by participants. The stress level of the improvised performance is less clear, however, the upward trend of ACTH and informal statement from one of the participants may perhaps indicate a higher level of stress experienced while improvising.

It is also recommended that future research account for potential effects of personality traits on proneness to flow during music production tasks. For instance, it has been observed that perfectionist tendencies frequently inhibit flow (Bruya, 2010). With a small sample size, these characteristics could disproportionately influence individual and group-level data. Screening participants with a personality inventory could prove helpful in accounting for such differences and allow for comparisons of flow experience of individuals with varying personality traits.

During this study, the researchers informally noted differences in participants' behavioral tendencies between conditions. For example, a higher frequency of social interaction cues, such as eye contact, were observed during the improvisation task. Behavioral analyses combined with 7–8 formal interview questions of the participants' subjective experience would provide greater insight on social flow and affiliation in standard and improvised group singing.

As stated previously, the current study tested our procedures using typically functioning student musicians. Future researchers might employ the use of music improvisation in dyadic and group settings with non-diagnosed populations (this could include both musicians and non-musicians) first to facilitate positive social interactions and elicit desired nonmusical responses (MacDonald and Wilson, 2014). Once an understanding of biochemistry and flow during singing for typically functioning people is obtained, clinical research with appropriate populations can be pursued. For purposes of translating the research to clinical application, future researchers may also consider structuring the music production tasks in a way that more accurately reflects the current practices of music therapists. For example, many clinical experiences entail the playing of instruments as opposed to singing. Clinicians may also manipulate instruments in such a way that clients, despite age or ability, are able to participate in an error-free music-making experience (improvising modally, for example) that results in the creation of an aesthetically pleasing sound. Collaboration between practicing clinicians and researchers will be important in progressing this line of research and gaining more insight into the facilitation, experience, and outcomes of social flow and affiliation in the context of group music-making.

### Conflict of interest statement

The authors declare that the research was conducted in the absence of any commercial or financial relationships that could be construed as a potential conflict of interest.

## References

[B1] Amico J. A. Ulbrecht J. S. Robinson A. G. (1987). Clearance studies of oxytocin in humans using radioimmunoassay measurements of the hormone in plasma and urine. J. Clin. Endocrinol. Metab. 6, 240–245. 10.1210/jcem-64-2-340 3793853

[B2] Anshel A. Kipper D. A. (1988). The influence of group singing on trust and cooperation. J. Music Ther. 25, 145–155. 10.1093/jmt/25.3.145

[B3] Bachen C. M. Raphael C. (2011). Social Flow and Learning in Digital Games: A Conceptual Model and Research Agenda. London: Springer.

[B4] Baker F. A. MacDonald R. A. R. (2013). Flow, identity, achievement, satisfaction and ownership during therapeutic songwriting experiences with university students and retirees. Musicae Scientiae, 17, 131–146. 10.1177/1029864913476287

[B5] Bakker A. B. Oerlemans W. Demerouti E. Slot B. B. Ali D. K. (2011). Flow and performance: A study among talented dutch soccer players. Psychol. Sport Exerc. 12, 442–450. 10.1016/j.psychsport.2011.02.003

[B6] Bartz J. A. Zaki J. Bolger N. Ochsner K. N. (2011). Social effects of oxytocin in humans: context and person matter. Trends Cogn. Sci. 15, 301–309. 10.1016/j.tics.2011.05.002 21696997

[B7] Bittman B. B. Berk L. S. Felten D. L. Westengard J. (2001). Composite effects of group drumming music therapy on modulation of neuroendocrine-immune parameters in normal subjects. Altern. Ther. Health Med. 7, 38. 11191041

[B8] Bruya B. (2010). Effortless Attention: A New Perspective in the Cognitive Science of Attention and Action. Cambridge, MA: MIT Press.

[B9] Carter C. S. (2014). Oxytocin pathways and the evolution of human behavior. Annu. Rev. Psychol. 65, 17–39. 10.1146/annurev-psych-010213-115110 24050183

[B10] Carter C. S. Pournajafi-Nazarloo H. Kramer K. M. Ziegler T. E. White-Traut R. Bello D.. (2007). Oxytocin: behavioral associations and potential as a salivary biomarker. Ann. N.Y. Acad. Sci. 1098, 312–322. 10.1196/annals.1384.006 17435137

[B11] Chanda M. L. Levitin D. J. (2013). The neurochemistry of music. Trends Cogn. Neurosci. 17, 179–193. 10.1016/j.tics.2013.02.007 23541122

[B12] Clift S. Morrison I. (2011). Group singing fosters mental health and wellbeing: findings from the East Kent “singing for health” network project. Ment. Health Soc. Inclusion 15, 88–97. 10.1108/20428301111140930

[B13] Clift S. Nicol J. Raisbeck M. Whitmore C. Morrison I. (2010). Group Singing, Wellbeing and Health: A Systematic Mapping of Research Evidence. Kent: Sidney De Haan Research Centre for Arts and Health, Canterbury Christ Church University.

[B14] Csikszentmihalyi M. (1975). Beyond Boredom and Anxiety. San Francisco, CA: Jossey-Bass Publishers.

[B15] Csikszentmihalyi M. (1997). Finding flow: The Psychology of Engagement with Everyday Life. New York, NY: Basic Books.

[B16] Csikszentmihalyi M. LeFevre J. (1989). Optimal experience in work and leisure. J. Pers. Soc. Psychol. 56, 815–822. 10.1037/0022-3514.56.5.815 2724069

[B17] Dai L. Carter C. S. Ying J. Bellugi U. Pournajafi-Nazarloo H. Korenberg J. R. (2012). Oxytocin and vasopressin are dysregulated in Williams Syndrome, a genetic disorder affecting social behavior. PLoS One 7:e38513. 10.1371/journal.pone.0038513 22719898 PMC3373592

[B18] Diaz F. M. (2013). Mindfulness, attention, and flow during music listening: an empirical investigation. Psychol. Music 41, 42–58. 10.1177/0305735611415144

[B19] Ditzen B. Schaer M. Gabriel B. Bodenmann G. Ehlert U. Heinrichs M.. (2009). Intranasal oxytocin increases positive communication and reduces cortisol levels during couple conflict. Biol. Psychiatry 65, 728–731. 10.1016/j.biopsych.2008.10.011 19027101

[B20] Edison H. (Composer), Hendricks J. (Lyricist). (1958). Centerpiece. [arr. Greg Jasperse, 2015].

[B21] Engeser S. (2012). Advances in Flow Research. New York, NY: Springer.

[B22] Evers S. Suhr B. (2000). Changes of the neurotransmitter serotonin but not of hormones during short time music perception. Eur. Arch. Psychiatry Clin. Neurosci. 250, 144–147. 10.1007/s004060070031 10941989

[B23] Fancourt D. Aufegger L. Williamon A. (2015). Low-stress and high-stress singing have contrasting effects on glucocorticoid response. Front. Psychol. 6:1242 10.3389/fpsyg.2015.01242 PMC4559645 26388794

[B24] Fancourt D. Ockelford A. Belai A. (2014). The psychoneuroimmunological effects of music: A systematic review and a new model. Brain Behav. Immun. 36, 15–26. 10.1016/j.bbi.2013.10.014 24157429

[B25] Feldman R. Gordon I. Schneiderman I. Weisman O. Zagoory-Sharon O. (2010). Natural variations in maternal and paternal care are associated with systematic changes in oxytocin following parent–infant contact. Psychoneuroendocrinology 35, 1133–1141. 10.1016/j.psyneuen.2010.01.013 20153585

[B26] Freeman W. (1998). A neurobiological role of music in social bonding, in The Origins of Music, eds Wallin N. L. Merker B. Brown S. (Cambridge, MA: MIT Press), 411–424.

[B27] Fritz B. S. Avsec A. (2007). The experience of flow and subjective well-being of music students. Horiz. Psychol. 17, 5–17.

[B28] Gerra G. Zaimovic A. Franchini D. Palladino M. Giucastro G. Reali N.. (1998). Neuroendocrine responses of healthy volunteers totechno-music: relationships with personality traits and emotional state. Int. J. Psychophysiol. 28, 99–111. 9506313 10.1016/s0167-8760(97)00071-8

[B29] Gibbs D. M. (1986). Vasopressin and oxytocin: hypothalamic modulators of the stress response: a review. Psychoneuroendocrinology 11, 131–139. 10.1016/0306-4530(86)90048-X 3018820

[B30] Grape C. Sandgren M. Hansson L. O. Ericson M. Theorell T. (2002). Does singing promote well-being?: An empirical study of professional and amateur singers during a singing lesson. Integr. Physiol. Behav. Sci. 38, 65–74. 10.1007/BF02734261 12814197

[B31] Grossman A. Perry L. Schally A. V. Rees L. Kruseman A. N. Tomlin S. (1982). New hypothalamic hormone, corticotropin-releasing factor, specifically stimulates the release of adrenocorticotropic hormone and cortisol in man. Lancet 319, 921–922. 6122767 10.1016/s0140-6736(82)91929-8

[B32] Hart E. Di Blasi Z. (2015). Combined flow in musical jam sessions: a pilot qualitative study. Psychol. Music 43, 275–290. 10.1177/0305735613502374

[B33] Heinrichs M. von Dawans B. Domes G. (2009). Oxytocin, vasopressin, and human social behavior. Front Neuroendocrinol. 30, 548–557. 10.1016/j.yfrne.2009.05.005 19505497

[B34] Herman J. P. (2012). Neural pathways of stress integration: relevance to alcohol abuse. Alcohol Res. 34, 441. 23584110 10.35946/arcr.v34.4.08 PMC3860392

[B35] Hytönen-Ng E. (2013). Experiencing “Flow” in Jazz Performance. Farnham: Ashgate.

[B36] Jackson S. A. Eklund R. C. (2002). Assessing flow in physical activity: The flow state scale-2 and dispositional flow scale-2. J. Sport Exerc. Psychol. 24, 133–150.

[B37] Jackson S. A. Eklund R. C. Martin A. J. (2010). The Flow Scales Instrument and Scoring Guide. Menlo Park, CA: Mind Garden Inc.

[B38] Jackson S. A. Martin A. J. Eklund R. C. (2008). Long and short measures of flow: The construct validity of the FSS-2, DFS-2, and new brief counterparts. J. Sport Exerc. Psychol. 30, 561. 18971512 10.1123/jsep.30.5.561

[B39] Jackson S. A. Thomas P. R. Marsh H. W. Methurst C. J. (2001). Relationships between flow, self-concept, psychological skills, and performance. J. Appl. Sport Psychol. 13, 129–153. 10.1080/104132001753149865

[B40] Kosfeld M. Heinrichs M. Zak P. J. Fischbacher U. Fehr E. (2005). Oxytocin increases trust in humans. Nature 435, 673–676. 10.1038/nature03701 15931222

[B41] Kreutz G. (2014). Does singing facilitate social bonding? Music Med. 6, 51–60.

[B42] Kreutz G. Bongard S. Rohrmann S. Hodapp V. Grebe D. (2004). Effects of choir singing or listening on secretory immunoglobulin A, cortisol, and emotional state. J. Behav. Med. 27, 623–635. 10.1007/s10865-004-0006-9 15669447

[B43] LaGasse A. B. (2013). Pilot and feasibility studies: application in music therapy research. J. Music Ther. 50, 304–320. 10.1093/jmt/50.4.304 25014669

[B44] Leslie J. S. Toni E. Z. Seth D. P. (2010). Social vocalizations can release oxytocin in humans. Proc. R. Soc. B Biol. Sci. 277, 2661–2666. 10.1098/rspb.2010.0567 20462908 PMC2982050

[B45] MacDonald R. A. Wilson G. B. (2014). Musical improvisation and health: a review. Psychol. Well Being 4, 1–18. 10.1186/s13612-014-0020-9

[B46] MacDonald R. Byrne C. Carlton L. (2006). Creativity and flow in musical composition: an empirical investigation. Psychol. Music 34, 292–306. 10.1177/0305735606064838

[B47] Martin W. L. Carter C. S. (2013). Oxytocin and vasopressin are sequestered in plasma, in 10th World Congress of Neurohypophyseal Hormones Abstracts (Bristol).

[B48] Mauri A. Volpe A. (1994). Stress mediators in the amniotic compartment in relation to the degree of fetal distress. Fetal Diagn. Ther. 9, 300–305. 10.1159/000263952 7818778

[B49] McCullough M. E. Churchland P. S. Mendez A. J. (2013). Problems with measuring peripheral oxytocin: can the data on oxytocin and human behavior be trusted? Neurosci. Biobehav. Rev. 37, 1485–1492. 10.1016/j.neubiorev.2013.04.018 23665533

[B50] Mugford A. L. (2004). Flow in a Team Sport Setting: Does Cohesion Matter? Ph.D. dissertation, University of Kansas.

[B51] Nilsson U. (2009). Soothing music can increase oxytocin levels during bed rest after open-heart surgery: a randomised control trial. J. Clin. Nurs. 18, 2153–2161. 10.1111/j.1365-2702.2008.02718.x 19583647

[B52] Pitts S. E. (2004). Everybody wants to be Pavoratti: The experience of music for performers and audience at a gilbert and sullivan festival. J. R. Music. Assoc. 129, 143–160. 10.1093/jrma/129.1.143

[B53] Rathunde K. (1997). Parent–Adolescent interaction and optimal experience. J. Youth Adolesc. 26, 669–689. 10.1023/A:1022344608624

[B54] Rogers S. E. (2013). Researching musical improvisation: Questions and challenges. Psychomusicology 23, 269–272. 10.1037/pmu0000027

[B55] Salanova M. Rodríguez-Sánchez A. M. Schaufeli W. B. Cifre E. (2014). Flowing together: A longitudinal study of collective efficacy and collective flow among workgroups. J. Psychol. 148, 435. 10.1080/00223980.2013.806290 24946388

[B56] Sandman C. A. George J. McCanne T. R. Nolan J. D. Kaswan J. Kastinj A. J. (1977). MSH/ACTH 4-10 influences behavioral and physiological measures of attention 1. J. Clin. Endocrinol. Metab. 44, 884–891. 10.1210/jcem-44-5-884 192754

[B57] Sandman C. A. George J. M. Nolan J. D. Van Riezen H. Kastin A. J. (1975). Enhancement of attention in man with ACTH/MSH 4–10. Physiol. Behav. 15, 427–431. 10.1016/0031-9384(75)90254-1 176674

[B58] Sawyer R. K. (2006). Group creativity: musical performance and collaboration. Psychol. Music 34, 148–165. 10.1177/0305735606061850

[B59] Takahashi T. Gribovskaja-Rupp I. Babygirija R. (2013). The Physiology of Love: Role of Oxytocin in Human Relationships, Stress Response, and Health. New York, NY: Nova Science Publishers Inc.

[B60] Uchino B. N. (2006). Social support and health: a review of physiological processes potentially underlying links to disease outcomes. J. Behav. Med. 29, 377–387. 10.1007/s10865-006-9056-5 16758315

[B61] Walker C. J. (2010). Experiencing flow: is doing it together better than doing it alone? J. Posit. Psychol. 5, 3–11. 10.1080/17439760903271116

[B62] Weijnen J. A. Slangen J. L. (1970). Effects of ACTH-analogues on extinction of conditioned behavior. Prog. Brain Res. 32, 221. 10.1016/S0079-6123(08)61538-1 4321521

[B63] Welch G. F. Himonides E. Saunders J. Papageorgi I. Sarazin M. (2014). Singing and social inclusion. Front. Psychol. 5:803. 10.3389/fpsyg.2014.00803 25120514 PMC4114289

[B64] Windle R. J. Kershaw Y. M. Shanks N. Wood S. A. Lightman S. L. Ingram C. D. (2004). Oxytocin attenuates stress-induced c-fos mRNA expression in specific forebrain regions associated with modulation of hypothalamo–pituitary–adrenal activity. J. Neurosci. 24, 2974–2982. 10.1523/JNEUROSCI.3432-03.2004 15044536 PMC6729832

[B65] Wrigley W. J. Emmerson S. B. (2013). The experience of the flow state in live music performance. Psychol. Music 41, 292–305. 10.1177/0305735611425903

[B66] Yalow R. S. Glick S. M. Roth J. Berson S. A. (1964). Radioimmunoassay of human plasma ACTH. J. Clin. Endocrinol. Metab. 24, 1219–1225. 10.1210/jcem-24-11-1219 14230021

